# The functions of the post-translational modifications in rheumatoid arthritis

**DOI:** 10.1186/ar3646

**Published:** 2012-02-09

**Authors:** Satoko Aratani, Naoko Yagishita, Teruhisa Kanazawa, Fukami Nakajima, Yoshihisa Yamano, Kusuki Nishioka, Toshihiro Nakajima

**Affiliations:** 1 Institute of Medical Science, Tokyo Medical University, Shinjuku-ku, Tokyo, 160-8402, Japan; 2 Institute of Medical Science, St. Marianna University School of Medicine, Kawasaki, Kanagawa, 216-8512, Japan

## 

Rheumatoid Arthritis (RA) is a chronic inflammatory joint disease and characterized by synovial hyperplasia. We previously cloned an E3 ubiquitin ligase, Synoviolin, as a regulatory factor of cell proliferation [1]. It suggested that endoplasmic reticulum (ER) associated degradation system (ERAD) via Synoviolin has important roles for overgrowth of synoviocytes [2,3]. Meanwhile, it is known that autoantibodies to citrullinated proteins are specific for RA and good markers for RA. Peptidyl-Arginine Deiminases 4 (PADI4) is identified as the RA-susceptible gene [3]. However functions of citrulinated proteins are unclear. In this study, we hypothesize that the accumulation of citrullinated proteins in RA synoviocytes could associate for ER stress and explore the crosstalk of ubiquitination and citrullination.
